# Global trends in low back pain and neck pain in the working population: implications for occupational health

**DOI:** 10.3389/fpubh.2025.1605072

**Published:** 2025-09-15

**Authors:** Yonghui Zhao, Jiqing Wang, Boya Zhao, Yingang Zhang

**Affiliations:** ^1^Department of Orthopedics, The First Affiliated Hospital of Xi'an Jiaotong University, Xi'an, China; ^2^Center for Reproductive Medicine, The First Affiliated Hospital of Zhengzhou University, Zhengzhou, China

**Keywords:** low back pain, neck pain, global burden database, occupational health, BAPC analysis

## Abstract

This study systematically evaluated the global burden of low back pain (LBP) and neck pain (NP) among individuals aged 20–65 from 1990 to 2021, utilizing data from the Global Burden of Disease (GBD) database. We analyzed incidence, prevalence, years lived with disability (YLDs), and age-standardized rates across 204 countries and regions, stratified by socio-demographic index (SDI). Key findings indicate a slight decline in LBP metrics but a mild increase in NP trends, with higher growth rates observed in females. Socioeconomic status significantly influenced these patterns, with developed countries showing lower YLDs increasing rate. The study highlights the need for gender-specific and region-tailored public health strategies, particularly focusing on women in lower SDI countries. These findings underscore the importance of targeted interventions to mitigate the growing burden of LBP and NP in an aging workforce increasingly engaged in office work.

## Introduction

Neck pain and low back pain are among the most prevalent musculoskeletal disorders in the working-age population worldwide, undermining quality of life and workforce productivity (1, 2). Occupational and lifestyle factors—including prolonged sitting, repetitive or heavy manual tasks, manual handling, and suboptimal ergonomics—are associated with low back and neck pain across both industrialized and agricultural settings (3, 4). Beyond persistent pain, these conditions contribute to decreased productivity and job absenteeism, underscoring the need for systematic, stratified analyses of their epidemiological trends (5–7).

The Global Burden of Disease (GBD) framework offers standardized, multi-source data to explore such heterogeneity (8). Although existing studies have used GBD data to examine the overall burden of musculoskeletal diseases, there is still a lack of systematic analysis on the global heterogeneity, socioeconomic drivers, and temporal evolution patterns of neck and low back pain specifically in the 20–65-year-old working-age group (9). In line with Eurostat’s definition of the core working-age population and prior research,^1^ we define individuals aged 20–65 years as the working-age population in this study (10, 11). Previous research has mostly focused on specific occupational groups (e.g., healthcare workers, drivers) or local regions (e.g., high-income countries), making it difficult to fully reveal the differences between these diseases across countries at different development levels and their association with population aging and lifestyle changes (12, 13). Moreover, insufficient attention has been paid to the social inequalities in disease burden (such as SDI stratification differences) and future trend predictions, limiting policymakers’ ability to optimize resource allocation from a macro perspective (14). Consequently, a globally comparable, finely stratified assessment targeting the working-age population is still lacking.

To bridge this gap, we drew on GBD 2021 data to quantify the incidence, prevalence, and years lived with disability (YLDs) attributable to neck pain and low back pain among adults aged 20–65 years across 204 countries and territories from 1990 to 2021. Unlike prior studies that focus on specific occupational groups or regions, our study provides a global perspective across socioeconomic and gender strata, offering actionable insights for policymakers. Worldwide estimates were disaggregated by SDI quintile, sex, and GBD region; Joinpoint regression detected temporal inflection points; a Bayesian age–period–cohort model projected trends to 2036 (15); and decomposition analysis partitioned the contributions of population aging, epidemiological transition, and population growth. To capture the dynamics of health inequality, frontier analysis and concentration indices identified countries and regions exhibiting excess burden even at comparable SDI levels. This integrative framework—combining spatiotemporal distribution, socioeconomic determinants, and forward projections—provides an evidence base for targeted occupational-health interventions, optimized resource allocation, and cross-regional collaboration, thereby filling a critical gap in the macro-epidemiological landscape of neck and low back pain among working-age adults.

Therefore, this study aims to systematically quantify the incidence, prevalence, and YLDs of LBP and NP among adults aged 20–65 years across 204 countries and territories from 1990 to 2021, examine temporal trends and SDI-stratified sex differences, and project future burdens to inform occupational-health policy.

## Methods

### Research design and data sources

This ecological study is based on data from the 2021 Global Burden of Disease Study (GBD 2021), which systematically analyzes the prevalence, incidence, and disability burden of low back pain (LBP) and neck pain (NP) among working-age adults (aged 20–65) across 204 countries and territories from 1990 to 2021. The GBD 2021 incorporates epidemiological data from population-based surveys, civil registration systems, hospital records, and published literature, using standardized protocols for data harmonization to ensure comparability across regions (16). Demographic and age structure data are sourced from the United Nations Population Division (UNPD), aligning with global population estimates. Data were obtained through the GBD Results Tool^2^ and reanalyzed according to the methods of GBD 2021.

### Case definition and data extraction

GBD 2021 defines LBP and NP as chronic pain conditions (with a duration of ≥3 months accompanied by functional disability) diagnosed based on symptoms and clinical assessment (2). Incidence, prevalence, and years lived with disability (YLDs) specific to the 20–65 age group were extracted for analysis. YLDs were calculated by multiplying the prevalence by disease-specific disability weights, with adjustments made for comorbidity effects (17). Given that LBP and NP predominantly contribute to non-fatal burden, this study focuses solely on YLDs. Countries were categorized into five groups based on the Socio-demographic Index (SDI) (low, lower-middle, middle, upper-middle, and high), and further stratified analyses were conducted according to the 21 GBD geographic regions.

### Data processing and age standardization

The GBD 2021 employs DisMod-MR 2.1, a Bayesian meta-regression tool, to internally validate and correct for biases across heterogeneous data sources. Age-standardized rates (ASRs) per 100,000 population were calculated using the GBD 2021 standard population structure, with uncertainty intervals (UIs) generated through 1,000 posterior samples (8). All analyses are limited to the 20–65 age group to focus on the working-age population.

This approach ensures robust estimation of prevalence, incidence, and YLDs for low back pain (LBP) and neck pain (NP), providing reliable insights specifically for the working-age demographic across the globe.

### Statistical analysis

The estimated annual percentage changes (EAPCs) of ASRs from 1990 to 2021 were calculated. Joinpoint regression analyses were performed with the Joinpoint Regression Program (version 4.9.1.0; National Cancer Institute, United States). The maximum number of joinpoints was restricted to five (< 6), permitting up to six linear segments, and the optimal model was selected by Monte-Carlo permutation tests with an overall significance level of *p* < 0.05. Annual percentage changes (APCs) and their 95% confidence intervals (CIs) were reported for each segment (18). The Das Gupta framework was applied to decompose the total change in YLDs into three dimensions: (1) population growth; (2) population aging (shifts in age structure); and (3) epidemiological change (dynamics of risk factors). Contributions of each dimension to global trends and SDI groups were quantified. Following the additive decomposition of Das Gupta (19), the absolute change in YLDs between two time points (t_1_, t_0_) is expressed as

$$\mathrm{\Delta Y}=\sum_{a}(P_{a,t1}-P_{a,t0})\bar{r_{a}}+\sum_{a}\bar{P_{a}}(r_{a,t1}-r_{a,t0})$$

where $\;P_{a,t}$ and $r_{a,t}$ denote the population size and age-specific YLD rate for age group *a* in year *t*, and bars indicate the mean of the two time points. The first term (further partitioned into growth and aging components via the stepwise-replacement algorithm) captures demographic effects, whereas the second term quantifies epidemiologic change.

To assess socioeconomic disparities in YLDs, the slope inequality index (SII) and concentration index (CI) were calculated. SII represents the absolute difference in YLD rates between the highest and lowest SDI quintiles, while CI measures distribution inequality, with negative values indicating a heavier burden in countries with higher SDI scores.

A Bayesian age–period–cohort (BAPC) model with a Poisson likelihood was implemented via the BAPC and INLA packages; age, period and cohort effects were specified as second-order random walks with penalized-complexity priors. Posterior medians and 95% credible intervals were obtained using integrated nested Laplace approximation, and the fitted model was used to project YLD counts and age-standardized rates from 2022 to 2036 (20).

### Software and ethics

Data analysis was conducted using R (version 4.3.1) and Stata (version 17.0). The study utilized publicly available de-identified data, thus was exempt from institutional ethics review (21).

## Results

### Global burden and trends of low back pain (LBP) and neck pain (NP)

As of 2021, low back pain (LBP) and neck pain (NP) remain leading causes of disability among individuals aged 20 to 65 globally. In 2021, there were 181.6 million new cases of LBP (age-standardized incidence rate: 4048.17 per 100,000), with 428.5 million prevalent cases (age-standardized prevalence: 9554.2 per 100,000), resulting in 48.84 million years lived with disability (YLDs) (age-standardized YLD rate: 1088.92 per 100,000). On the other hand, NP had 32.56 million new cases (incidence rate: 726.02 per 100,000), with 159 million prevalent cases (prevalence: 3359.13 per 100,000), contributing to 15.91 million YLDs (YLD rate: 354.62 per 100,000). From 1990 to 2021, LBP showed slight decreases in incidence (estimated annual percentage change [EAPC] = −0.05%), prevalence (EAPC = −0.06%), and YLDs (EAPC = −0.06%); conversely, NP experienced minor increases in incidence (EAPC = +0.12%), prevalence (EAPC = + 0.11%), and YLDs (EAPC = +0.18%) (Tables 1, 2).

**Table 1:** Incidence, prevalence, YLDs, and age-standardized YLD rates of working-age low back pain in 2021, with EAPC from 1990 to 2021, globally and by GBD region.

Locations	Number of incident cases (95%UIs)	Age-standardized rate of incidence per 100,000 (95%UIs)	Estimated annual percentage change of incidence per 100,000 from 1990 to 2021 (95%CI)		Number of prevalent cases (95%UIs)	Age-standardized rate of prevalence per 100,000 (95%UIs)	Estimated annual percentage change of prevalence per 100,000 from 1990 to 2021 (95%CI)	Number of YLDs (95%UIs)	Age-standardized rate of YLDs per 100,000 (95%UIs)	Estimated annual percentage change of YLDs per 100,000 from 1990 to 2021 (95%CI)
Global	181572558.01 (126582661.14–248120331.47)	4048.17 (2822.17–5531.85)	−0.05 (−0.11–0)		428548876.15 (312713838.26–572519896.96)	9554.52 (6971.97–12764.36)	−0.06 (−0.11–0)	48841562.4 (30282587.17–72683781.87)	1088.92 (675.15–1620.49)	−0.06 (−0.11–0)
Female	111664664.83 (78146252.2–152135143.67)	4999.17 (3498.57–6811.02)	0.01 (−0.06–0.07)		266488931.2 (194564886.85–354001362.95)	11930.58 (8710.58–15848.47)	−0.02 (−0.08–0.05)	30140418.2 (18725837.19–44776368.22)	1349.37 (838.35–2004.62)	−0.02 (−0.08–0.05)
Male	69907893.19 (48497000.93–95649402.64)	3104.76 (2153.85–4247.99)	−0.16 (−0.2–−0.12)		162059944.95 (117599262.54–217837900.26)	7197.42 (5222.83–9674.63)	−0.14 (−0.18–−0.11)	18701144.2 (11557851.35–28001732.26)	830.56 (513.31–1243.62)	−0.14 (−0.18–−0.1)
High SDI	36657885.11 (26655995.78–48530547.86)	5291.21 (3847.47–7016.15)	−0.04 (−0.08–0)		88603854.71 (68023829.32–113806708.26)	12730.6 (9776.9–16345.92)	−0.05 (−0.09–−0.01)	10098778.2 (6499971.06–14671177.17)	1452.3 (932.94–2110.37)	−0.06 (−0.09–−0.02)
High-middle SDI	35343343.08 (24552719.27–48353381.39)	4052.25 (2810.42–5554.6)	−0.12 (−0.19–−0.05)		83351787.56 (60294239.91–111665319.67)	9533.71 (6891.68–12776.58)	−0.13 (−0.2–−0.05)	9546760.07 (5894711.58–14273493.74)	1092.82 (673.8–1636.09)	−0.11 (−0.19–−0.04)
Middle SDI	52724758.59 (36243958.36–72555916.4)	3540.35 (2432.7–4872.93)	0.08 (0.01–0.1)		123196452.28 (87977127.51–166508611.8)	8268.42 (5904.74–11174.21)	0.09 (0.02–0.17)	14077003.66 (8630157.62–21100537.26)	944.69 (579.01–1416.39)	0.09 (0.02–0.16)
Low-middle SDI	39170033.6 (26889205.67–54072939.43)	4017.52 (2763.35–5533.08)	0.08 (0.02–0.13)		92189449.9 (65910717.31–124661876.37)	9497.44 (6792.53–12843.53)	0.09 (0.03–0.15)	10446706.67 (6387024.75–15724426.8)	1074.88 (658.81–1615.76)	0.1 (0.04–0.15)
Low SDI	17510580.85 (12020718.62–24194567.81)	4040.97 (2783.93–5560.85)	0.08 (0.05–0.11)		40806010.57 (29329835.15–54916770.93)	9498.93 (6824.92–12789.79)	0.08 (0.05–0.11)	4626464.02 (2841695.76–6930431.21)	1074.97 (662.7–1606.18)	0.1 (0.07–0.14)
Central Europe, Eastern Europe, and Central Asia	15756630.8 (11035796.81–21442831.16)	5841.77 (4081.48–7966.1)	0.16 (0.14–0.17)		38206227.67 (27785304.29–50717436.85)	14117.1 (10259.85–18767.28)	0.16 (0.14–0.18)	4346365.15 (2710230.77–6454449.17)	1608.57 (1001.5–2391.12)	0.17 (0.15–0.19)
Central Asia	2689696.03 (1862955.57–3686900.26)	4952.28 (3431.67–6783.87)	0.26 (0.25–0.28)		6273333.97 (4523957.27–8389909.24)	11589.86 (8359.01–15507.05)	0.3 (0.28–0.32)	718625.57 (444377.54–1070658.54)	1327.14 (821.23–1977.91)	0.3 (0.28–0.32)
Central Europe	5015271.79 (3526649.93–6796550.15)	6685.96 (4686.47–9083.13)	0.15 (0.14–0.16)		12585741.63 (9179005.65–16620578.25)	16667.02 (12148.28–22025.37)	0.15 (0.14–0.16)	1439095.39 (897204.66–2137342.76)	1909.22 (1188.12–2838.31)	0.17 (0.16–0.18)
Eastern Europe	8051662.99 (5642478.73–10948395.52)	5750.65 (4015.76–7845.6)	0.23 (0.19–0.26)		19347152.08 (14098450.5–25709205.99)	13764.5 (10016.7–18326.9)	0.24 (0.2–0.28)	2188644.19 (1367577.83–3251574.97)	1560.82 (972.41–2322.69)	0.25 (0.21–0.29)
High-income	36910676.27 (26654796.82–49079121.03)	5468.45 (3951.56–7284.45)	0.06 (0.02–0.1)		88899248.73 (67725768.57–114727699.75)	13103.01 (9994.37–16892.41)	0.05 (0.01–0.09)	10127033.76 (6485721.81–14758904.26)	1494.04 (954.96–2177.12)	0.04 (0–0.08)
Australasia	1176876.6 (820445.1–1600466.27)	6268.83 (4372.1–8539.32)	0.02 (−0.01–0.04)		2884813.01 (2087053.52–3854006.95)	15334.23 (11096.09–20493.34)	−0.03 (−0.06–0.01)	328498.01 (204053.38–491875.69)	1746.37 (1084.8–2615.69)	−0.02 (−0.05–0.01)
High-income Asia Pacific	6365974.07 (4436753.97–8681632.72)	5474.15 (3807.81–7485.06)	−0.05 (−0.08–−0.03)		15464057.1 (11169677.93–20726150.2)	13208.53 (9541.08–17686.04)	−0.08 (−0.11–−0.05)	1787781.59 (1104589.54–2674123.92)	1528.19 (941.76–2285.86)	−0.07 (−0.1–−0.05)
High-income North America	13151765.58 (9948222.08–16742447.98)	5849.79 (4427.03–7452.73)	0.01 (−0.06–0.08)		31755631.33 (25906247.66–38717130.83)	14053.95 (11469.6–17131.48)	0.03 (−0.05–0.1)	3578507.07 (2401737.31–4997756.65)	1586.24 (1062.56–2216.43)	0 (−0.07–0.07)
Southern Latin America	2192281.66 (1526408.46–2995472.86)	5459.58 (3801.34–7460.25)	0.17 (0.13–0.21)		5230311.2 (3778756.51–6989702.29)	13021.97 (9406.13–17401.96)	0.18 (0.14–0.23)	595666.8 (366660.37–889780.76)	1483.3 (913.07–2215.1)	0.18 (0.14–0.23)
Western Europe	14023778.36 (9728510.13–19160787.75)	5076.8 (3516.14–6954.48)	0.12 (0.09–0.16)		33564436.08 (24260749.2–44973826.63)	12104.22 (8753.47–16209.47)	0.11 (0.08–0.14)	3836580.28 (2372626.4–5711068.61)	1384.56 (854.49–2064.33)	0.11 (0.08–0.14)
Latin America and Caribbean	14966959.64 (10341152.4–20555621.55)	4315.78 (2981.99–5926.1)	0.2 (0.17–0.22)		36070635.87 (26005620.31–48570508.81)	10405.93 (7501.59–14015.17)	0.22 (0.19–0.25)	4090133.52 (2519633.68–6107668.33)	1179.72 (726.86–1761.37)	0.22 (0.2–0.25)	
Andean Latin America	1160434.49 (802327.42–1596611.33)	3236.94 (2239.61–4450.03)	0.25 (0.22–0.28)		2659849.63 (1914361.41–3584630.47)	7434.72 (5349.43–10023.33)	0.25 (0.22–0.29)	304274.02 (186665.71–455502.8)	849.99 (522.03–1271.34)	0.26 (0.22–0.29)	
Caribbean	924088.63 (643921.02–1262478.22)	3370.88 (2349.86–4605.62)	0.19 (0.17–0.21)		2144190.38 (1558461.96–2859423.23)	7819.42 (5683.58–10427.03)	0.21 (0.19–0.23)	243875.83 (151579.93–363073.23)	889.48 (552.92–1324)	0.2 (0.18–0.23)	
Central Latin America	6053605.75 (4167598–8321020.27)	4187.3 (2883.03–5754.29)	0.21 (0.16–0.26)		14562459.87 (10481354.21–19653674.5)	10082.54 (7254.78–13610.26)	0.23 (0.17–0.29)	1657619.1 (1020099.46–2479366.15)	1147.4 (706.15–1715.83)	0.23 (0.17–0.28)	
Tropical Latin America	6828830.77 (4718601.4–9388548.83)	4912.3 (3392.35–6754.85)	0.18 (0.17–0.2)		16704135.99 (12034938.81–22484984.84)	12018.69 (8657.57–16183.79)	0.21 (0.19–0.23)	1884364.56 (1162387.82–2816484.79)	1355.73 (836.22–2026.99)	0.22 (0.2–0.23)	
North Africa and Middle East	30860926.61 (21236997.31–42452478.62)	4499.09 (3094.59–6181.62)	0.1 (0.08–0.11)		74228580.65 (53531707.44–99674664.95)	10830.39 (7799.65–14562.68)	0.1 (0.08–0.12)	8435497.61 (5181132.23–12644375.99)	1229.2 (755.71–1841.49)	0.09 (0.07–0.11)	
South Asia	75224915.68 (51552568.33–104159692.85)	3866.21 (2654.61–5340.35)	−0.05 (−0.15–0.06)		175009006.19 (124847167.83–236670213.4)	9038.81 (6452.71–12219.17)	−0.06 (−0.17–0.06)	19749963.76 (12086268.92–29778030.2)	1018.63 (624.9–1533.93)	−0.05 (−0.16–0.06)	
Southeast Asia, East Asia, and Oceania	43813626.74 (30345655.31–60049104.22)	3064.74 (2120.43–4205.83)	−0.05 (−0.14–0.04)	3064.74 (2120.43–4205.83)	101357174.15 (72906520.94–136967034.71)	7072.57 (5087.14–9556.22)	−0.03 (−0.13–0.08)	11710303.27 (7187682.31–17599420.09)	817.46 (501.33–1229.88)	−0.02 (−0.13–0.08)	
East Asia	30242827.25 (21022279.58–41406252.92)	2980.87 (2070.07–4089.8)	−0.17 (−0.29–−0.05)		69563798.54 (50159732.23–93809592.92)	6841.46 (4930.62–9227.34)	−0.16 (−0.3–−0.03)	8047324.04 (4956604.42–12088777.77)	792.1 (487.28–1191.74)	−0.16 (−0.29–−0.02)	
Oceania	228031.21 (155306.39–313974.99)	3528.68 (2409.99–4846.56)	0.24 (0.23–0.25)		526951.48 (374424.74–715162.21)	8207.18 (5829.99–11141.51)	0.28 (0.26–0.29)	60279.74 (36562.97–90530.42)	937.11 (570.08–1406.21)	0.27 (0.26–0.29)	
Southeast Asia	13342768.28 (9135478.69–18422931.34)	3215.23 (2201.18–4438.59)	0.21 (0.19–0.23)		31266424.13 (22307225.21–42321880.56)	7538.05 (5378.06–10204.58)	0.25 (0.23–0.28)	3602699.48 (2195857.07–5417270.21)	868.41 (529.34–1305.59)	0.27 (0.25–0.29)	
Sub-Saharan Africa	17081743.43 (11730371.24–23641468.14)	3957.22 (2727.14–5458.76)	0.17 (0.17–0.18)		39396796.3 (28340961.05–53158472.52)	9206.3 (6616.47–12430.27)	0.19 (0.18–0.2)	4474996.02 (2744292.52–6726539.63)	1043.96 (642.56–1564.43)	0.21 (0.2–0.22)	
Central Sub-Saharan Africa	2175515.19 (1487864.19–3010065)	4159.52 (2853.55–5732.24)	0.14 (0.13–0.15)		5035789.59 (3597917–6816711.18)	9704.63 (6932.12–13127.9)	0.14 (0.13–0.16)	569627.43 (349760.2–858740.48)	1096.1 (675.93–1647.41)	0.18 (0.17–0.19)	
Eastern Sub-Saharan Africa	6535706.77 (4485207.71–9031236.64)	4108.17 (2830.88–5657.02)	0.14 (0.13–0.15)		15115936.51 (10867509.37–20387075.09)	9595.87 (6887.89–12958.85)	0.14 (0.13–0.16)	1719301.41 (1053818.05–2578725.42)	1089.54 (670.84–129.18)	0.17 (0.16–0.18)	
Southern Sub-Saharan Africa	1452330.83 (998766.7–2001713.57)	3554.86 (2447.82–4889.16)	0.19 (0.17–0.22)		3333821.02 (2378302.34–4505240.65)	8198.21 (5849.59–11085.12)	0.22 (0.19–0.25)	372746.86 (228911.08–558453.25)	915.2 (563.21–1369.73)	0.18 (0.15–0.2)	
Western Sub-Saharan Africa	6918190.64 (4751678.72–9573959.63)	3859.19 (2659.35–5319.39)	0.19 (0.16–0.22)		15911249.18 (11438674.51–21479140.9)	8954.7 (6435.52–12091.51)	0.22 (0.19–0.25)	1813320.33 (1110477.93–2724869.53)	1019.08 (626.86–1526.83)	0.24 (0.21–0.27)	

SG, Senile Gout; SDI, Sociodemographic Index; GBD, Global Burden of Disease, Injuries, and Risk Factors Study; UIs, Uncertainty Intervals; CI, Confidence Intervals.

**Table 2:** Incidence, prevalence, YLDs, and age-standardized YLD rates of working-age neck pain in 2021, with EAPC from 1990 to 2021, globally and by GBD region.

Locations	Number of incident cases (95%UIs)	Age-standardized rate of incidence per 100000 (95%UIs)	Estimated annual percentage change of incidence per 100000 from 1990 to 2021 (95%CI)	Number of prevalent cases (95%UIs)	Age-standardized rate of prevalence per 100000 (95%UIs)	Estimated annual percentage change of prevalence per 100000 from 1990 to 2021 (95%CI)	Number of YLDs (95%UIs)	Age-standardized rate of YLDs per 100000 (95%UIs)	Estimated annual percentage change of YLDs per 100000 from 1990 to 2021 (95%CI)
Global	32564130.88 (14532304.07–57425217.6)	726.02 (324–1280.3)	0.12 (0.05–0.19)	158740727.76 (92814880.22–253608305.93)	3539.13 (2069.31–5654.21)	0.18 (0.1–0.25)	15905760.36 (8370215–27776496.83)	354.62 (186.61–619.28)	0.17 (0.1–0.25)
Female	18896200.76 (8451153.26–33380557.45)	845.97 (378.35–1494.43)	0.17 (0.11–0.24)	95121301.82 (55552997.02–150567736.7)	4258.54 (2487.08–6740.85)	0.24 (0.17–0.32)	9461023.03 (5004005.85–16431920.31)	423.57 (224.03–735.65)	0.24 (0.16–0.31)
Male	13667930.12 (6106995.69–24127005.46)	607.02 (271.22–1071.53)	0.04 (−0.03–0.11)	63619425.94 (36791333.88–102999193.45)	2825.47 (1633.98–4574.41)	0.07 (−0.01–0.14)	6444737.33 (3344880.97–11307789.57)	286.22 (148.55–502.2)	0.07 (−0.01–0.14)
High SDI	4537985.96 (2032716.29–7998405.1)	677.48 (302.29–1190.91)	0.04 (−0.04–0.12)	25408334.77 (15131318.08–39473658.75)	3694.37 (2190.99–5756.93)	0.08 (−0.01–0.17)	2540781.88 (1357375.58–4374248.33)	370.46 (197.16–639.34)	0.06 (−0.03–0.15)
High-middle SDI	6664277.82 (2980843.91–11826898.67)	781.73 (348.23–1383.71)	0.16 (0.14–0.18)	33171528.28 (19436269.43–52901920.91)	3807.5 (2222.16–6086.72)	0.2 (0.16–0.24)	3332268.97 (1762621.43–5829823.02)	383.45 (202.02–671.41)	0.2 (0.16–0.24)
Middle SDI	11562862.39 (5176022.26–20388281.05)	778.22 (347.9–1370.48)	0.14 (0.09–0.19)	54431196.48 (31435036.93–87777048.65)	3647.84 (2104.62–5886.91)	0.25 (0.19–0.31)	5463534.55 (2858476.88–9583188.33)	366.28 (191.47–642.39)	0.24 (0.19–0.3)
Low-middle SDI	6517330.49 (2891281.66–11469599.48)	649.16 (289.06–1145.52)	0.09 (−0.07–0.25)	30422340.01 (17696639.76–48986610.11)	3099.93 (1809.18–4982.37)	0.21 (0.04–0.38)	3039350.87 (1585912.19–5335117.19)	308.81 (161.66–541.32)	0.21 (0.04–0.38)
Low SDI	3255215.63 (1441254.59–5733281.63)	702.73 (313.8–1244.85)	0.13 (0.04–0.23)	15173193.94 (8772889.66–24375578.9)	3447.33 (2006.31–5500.08)	0.26 (0.16–0.36)	1516377.04 (786085.33–2642911.58)	342.78 (178.74–594.29)	0.27 (0.17–0.38)
Central Europe, Eastern Europe, and Central Asia	1957037.57 (868899.2–3472274.22)	760.7 (335.42–1348.6)	0.06 (0.05–0.07)	9616941.89 (5542456.45–15368268.92)	3683.14 (2113.66–5902.91)	0.14 (0.13–0.14)	961022.9 (498930.37–1676890.24)	369.32 (191.17–647.02)	0.14 (0.14–0.15)
Central Asia	365642.77 (161128.51–646774.63)	667.46 (294.48–1181.32)	0.08 (0.07–0.08)	1848690.98 (1056406.42–2965450.19)	3390.14 (1938.07–5434.34)	0.16 (0.16–0.17)	186267.34 (94898.76–328487.89)	341.35 (174.14–602.01)	0.16 (0.16–0.16)
Central Europe	516926.18 (230709.22–918607.65)	723.42 (319.61–1282.53)	0.11 (0.1–0.12)	2599810.28 (1501922.63–4136732.34)	3560.78 (2045.15–5688.38)	0.18 (0.17–0.18)	260637.15 (135277.92–455933.55)	358.48 (185.38–630.25)	0.18 (0.18–0.19)
Eastern Europe	1074468.62 (477641.72–1906245.3)	821.55 (362.19–1456.73)	0.1 (0.09–0.11)	5168440.64 (2977418.29–8292911.68)	3881.18 (2224.76–6249.84)	0.16 (0.16–0.16)	514118.41 (265134.73–901864.23)	387.96 (199.09–683.9)	0.17 (0.16–0.17)
High-income	4156977.53 (1821740.86–7390779.02)	639.89 (279.29–1133.28)	−0.12 (−0.22–−0.02)	24594254.72 (14496549.98–38323734.53)	3667.2 (2150.71–5734.21)	−0.07 (−0.18–0.05)	2457943.88 (1295980.42–4245500.73)	367.6 (193.11–636.02)	−0.08 (−0.19–0.04)
Australasia	66156.54 (28374.97–119565.51)	358.1 (153.01–646.53)	0.03 (−0.03–0.1)	345336.79 (196759–557402.82)	1841.4 (1048.66–2977.19)	0.1 (0.02–0.17)	34532.99 (17597.52–60841.14)	184.46 (93.88–325.72)	0.09 (0.02–0.17)
High-income Asia Pacific	708307.34 (311599.13–1263488.44)	630.84 (275.96–1119.35)	−0.02 (−0.03–−0.01)	3728955.08 (2162076.46–5979278.72)	3219.04 (1859.44–5174.18)	0.09 (0.07–0.1)	377110.84 (194506.96–661373.49)	326.78 (167.88–575.24)	0.08 (0.07–0.09)
High-income North America	1431548.67 (633950.39–2534845.63)	660.65 (291.88–1165.46)	−0.23 (−0.44–−0.02)	7626360.28 (4373131.22–12213807.83)	3455.07 (1979.04–5533.61)	−0.18 (−0.38–0.02)	756006.23 (389332.88–1341246.66)	343.56 (176.76–609.23)	−0.2 (−0.4–0)
Southern Latin America	215462.01 (92007.31–382220.29)	537.42 (229.35–953.75)	0.06 (0.06–0.07)	1209841.34 (702604.82–1933029.01)	3015.08 (1750.83–4816.91)	0.17 (0.16–0.17)	121242.72 (61728.26–212567.58)	302.22 (153.88–530.15)	0.15 (0.15–0.16)
Western Europe	1735502.97 (749415.89–3121984.44)	659.64 (284.44–1180.75)	−0.07 (−0.15–0.01)	11683761.24 (7004376.55–17981251.25)	4243.62 (2528.12–6567.24)	−0.01 (−0.13–0.1)	1169051.09 (621940.62–1984975.13)	425.97 (225.67–725.3)	−0.01 (−0.13–0.1)
Latin America and Caribbean	2811411.28 (1248230.72–4936071.73)	803.86 (356.89–1412.87)	−0.02 (−0.06–0.02)	13630593.1 (7852102.92–22015177.77)	3915.13 (2256.56–6321.71)	0.04 (−0.01–0.09)	1363877.6 (708335.8–2388549.98)	391.52 (203.48–685.86)	0.03 (−0.02–0.08)
Andean Latin America	261856.8 (114171.54–461591.81)	703.97 (308.06–1243.74)	0.04 (0.04–0.05)	1322512.91 (756456.94–2138811.58)	3615.46 (2072.43–5,838)	0.13 (0.13–0.13)	133354.74 (68420.95–236523.44)	363.82 (187–644.81)	0.13 (0.12–0.13)
Caribbean	192846.58 (84199.84–340983.89)	704.11 (308.08–1243.95)	0.04 (0.04–0.04)	990581.44 (567363.3–1600887.22)	3616.19 (2072.88–5839.02)	0.13 (0.13–0.13)	99265.83 (51377.33–175728.37)	362.38 (187.63–641)	0.12 (0.11–0.12)
Central Latin America	1163415.71 (517539.41–2046752.78)	796.22 (354.47–1402.4)	0.05 (0.05–0.06)	5680946.86 (3278554.1–9136788.8)	3913.99 (2261.27–6289.62)	0.13 (0.12–0.13)	570093.13 (295715.56–996706.59)	392.41 (203.78–685.94)	0.11 (0.11–0.12)
Tropical Latin America	1193292.2 (529294.3–2103118.04)	858.28 (380.11–1513.96)	−0.12 (−0.23–−0.02)	5636551.89 (3223891.31–9210425.27)	4057.87 (2319.91–6635.76)	−0.09 (−0.22–0.03)	561163.9 (288657.06–989741.46)	404.02 (207.78–713.43)	−0.09 (−0.22–0.03)
North Africa and Middle East	7279195.22 (3248089.86–12852573.37)	1041.31 (463.53–1844.02)	0.12 (0.11–0.13)	37637955.7 (21978002.65–59309411.86)	5557.31 (3251.02–8757.25)	0.25 (0.25–0.26)	3741631.98 (1955620.73–6444609.97)	551.21 (288.71–949.81)	0.24 (0.23–0.25)
South Asia	10180665.76 (4526994.26–17950279.8)	508.89 (227.05–898.7)	−0.08 (−0.5–0.35)	44484752.11 (25342866.47–72952540.08)	2258.67 (1288.69–3701.36)	−0.01 (−0.47–0.46)	4431233.01 (2284454.8–7916637.82)	224.32 (115.84–400.44)	0 (−0.46–0.46)
Southeast Asia, East Asia, and Oceania	11054468.45 (5016997.28–19546669.98)	783.93 (355.38–1381.15)	0.22 (0.21–0.22)	51702374.94 (29875035.61–83610039.15)	3600.13 (2076.94–5828.79)	0.33 (0.31–0.34)	5223079.2 (2731951.37–9221932.96)	364.35 (190.11–642.45)	0.32 (0.31–0.33)
East Asia	7926603.17 (3610242.73–14048984.19)	801.41 (364.87–1412.89)	0.23 (0.22–0.24)	37142850.65 (21481491.59–60172034.04)	3653.3 (2109.1–5925.79)	0.34 (0.32–0.35)	3752126.12 (1957699.63–6635160.96)	370.11 (192.48–652.96)	0.33 (0.32–0.35)
Oceania	47042.52 (20863.92–83416.8)	704.89 (313.08–1255.23)	0.18 (0.17–0.18)	224110.17 (128239.97–358488.98)	3492.05 (2007.71–5568.14)	0.29 (0.28–0.3)	22518.13 (11444.88–39612.21)	349.35 (178.6–613.68)	0.29 (0.28–0.29)
Southeast Asia	3080822.76 (1382349.29–5438002.85)	739.32 (331.29–1305.5)	0.19 (0.19–0.2)	14335414.13 (8220724.52–23257596.29)	3453.39 (1980.66–5604.05)	0.29 (0.28–0.29)	1448434.95 (747901.17–2556930.45)	348.78 (180.15–615.93)	0.29 (0.29–0.3)
Sub-Saharan Africa	3854305.56 (1716861.9–6797868.86)	826.69 (371.26–1468.65)	0.17 (0.15–0.18)	18135209.2 (10493243.12–29103501.33)	4120.93 (2398.99–6571.91)	0.31 (0.3–0.32)	1813404.29 (937928.82–3157023.66)	410 (213.54–710.25)	0.32 (0.31–0.33)
Central Sub-Saharan Africa	423628.09 (185727.22–753776.13)	747.73 (330.09–1336.54)	0.09 (0.08–0.1)	2065252.45 (1186208.95–3312427.92)	3859.68 (2231.95–6140.93)	0.21 (0.2–0.21)	205665.86 (105182.41–360201.05)	382.55 (196.74–668.28)	0.23 (0.23–0.24)
Eastern Sub-Saharan Africa	1172046.65 (523448.76–2060891.53)	688.28 (310.02–1221.05)	0.13 (0.1–0.17)	5264364.28 (3021598.97–8547419.2)	3282.94 (1895.88–5297.22)	0.25 (0.21–0.29)	527910.83 (271486.98–935257.01)	327.4 (169.37–578.14)	0.27 (0.23–0.31)
Southern Sub-Saharan Africa	389995.86 (174613.53–690069.32)	896.3 (401.89–1594.75)	0.12 (0.1–0.15)	1816239.55 (1050417.22–2932442.71)	4302.6 (2495.04–6942.06)	0.23 (0.21–0.24)	178,929 (92400.3–312430.83)	422.57 (219.08–738.25)	0.18 (0.17–0.19)
Western Sub-Saharan Africa	1868634.97 (832864.47–3289264.73)	957.46 (430.62–1694.6)	0.21 (0.2–0.23)	8989352.92 (5237259.37–14354421.62)	4896.23 (2874.09–7766.62)	0.38 (0.37–0.39)	900898.6 (465733.49–1566105.72)	488.29 (254.03–844.34)	0.39 (0.38–0.4)

SG, Senile Gout; SDI, Sociodemographic Index; GBD, Global Burden of Disease, Injuries, and Risk Factors Study; UIs, Uncertainty Intervals; CI, Confidence Intervals.

**Figure 1:**
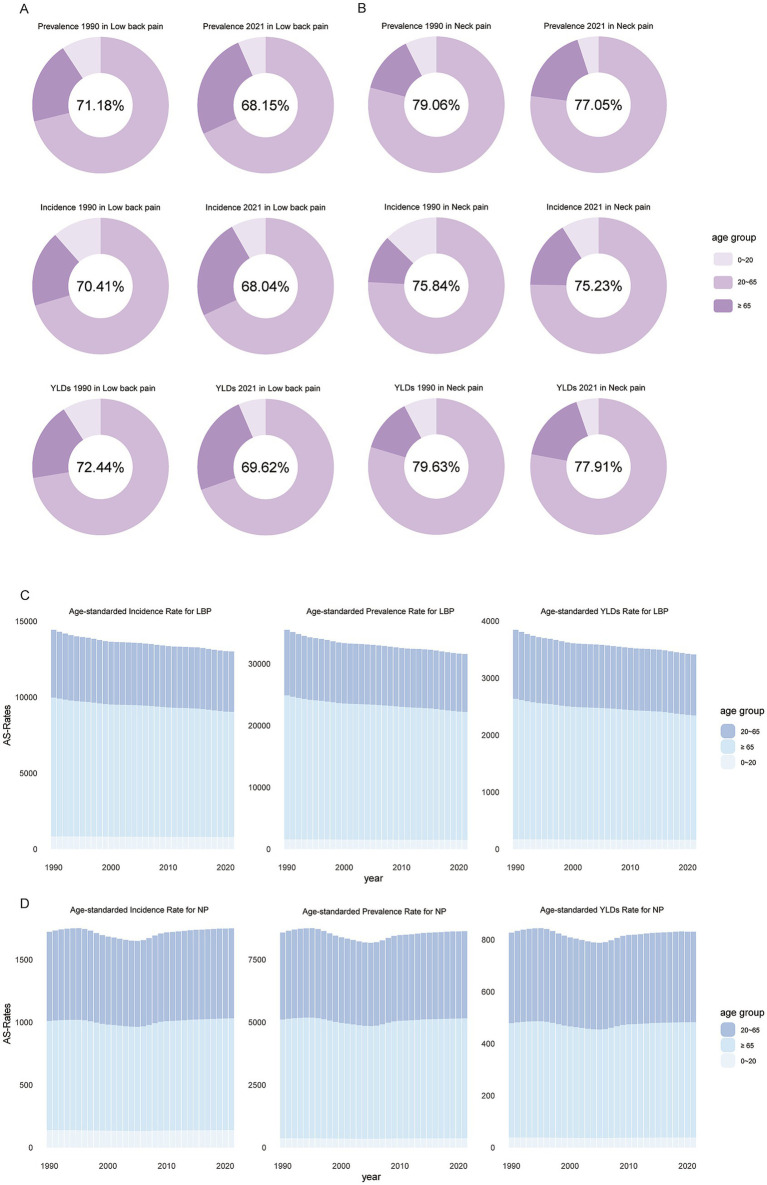
Global proportion of incident cases, prevalent cases, and YLDs (number) for neck pain and low back pain among individuals aged 20–65 **(A,B)**, and the trend of age-standardized changes over time **(C,D)**. YLDs, years lived with disability.

Regarding NP, from 1990 to 2021, there was a slight decrease in incidence, prevalence, and number of YLDs. Similar to NP, LBP exhibited a comparable trend. After age standardization, the overall trend for LBP remained on a decline. However, individuals over 65 showed significantly higher rates of incidence, prevalence, and YLDs for LBP, indicating that after adjusting for population proportions and changes, older adults are more susceptible to LBP (Figures 1A,B). For NP, around 2005 saw the lowest points for incidence, prevalence, and YLDs. Post-2007, these rates gradually increased among those over 65 and among the 20–65 age, while growth was less noticeable under 20s (Figures 1C,D).

Overall, People over 65 have higher incidence rates for both conditions. Individuals aged 20 to 65 account for the majority of LBP and NP cases, and prevention within the working-age population (aged 20–65) is crucial for control the NP and LBP, making this demographic a focal point for subsequent epidemiological analysis.

### Age-stratified trends in incidence, prevalence, and YLDs

**Figure 2:**
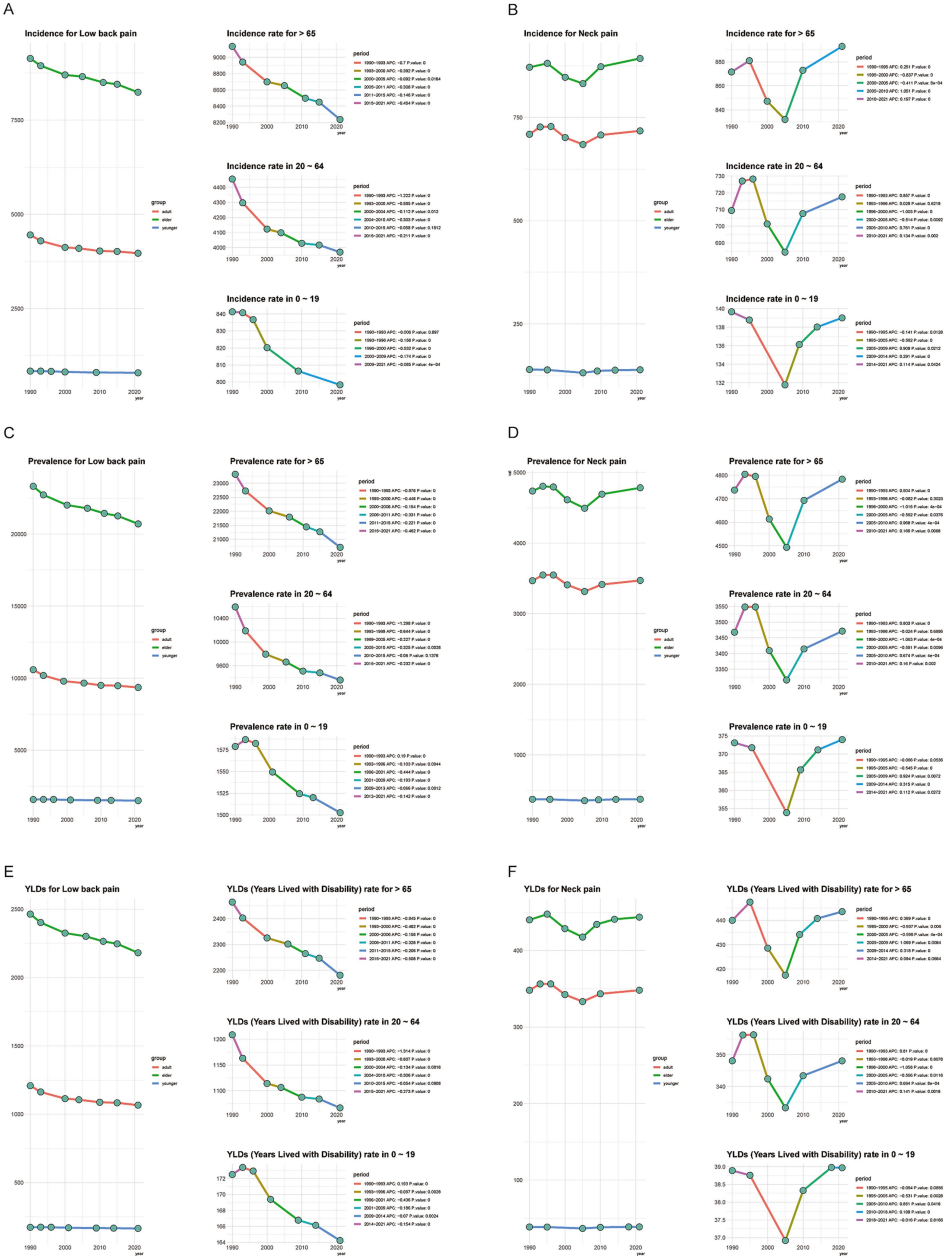
Joinpoint regression analysis of neck pain (NP) and low back pain (LBP) by age group, 1990–2021 **(A–F)**. The x-axis shows calendar year (1990–2021). The y-axis shows age-standardized rates per 100,000 (incidence, prevalence, and YLD rate). Each curve denotes one age group: 0–19 years, 20–64 years, and ≥65 years. Joinpoints indicate statistically significant changes in trend; APCs are reported for each segment (Monte-Carlo permutation tests, *p* < 0.05). APC, annual percentage change; YLDs, years lived with disability.

Joinpoint regression analysis (Figure 2) revealed differentiated trajectories of low back pain (LBP) and neck pain (NP) across different age groups from 1990 to 2021. For LBP, incidence rates showed significant declines in individuals aged ≥65 and those aged 20–64, with the most substantial decreases occurring between 1990 and 1993, reaching −0.7 and −1.222, respectively. The 0–19 age group maintained a relatively low incidence rate throughout the period, with a minor decrease. Similar trends were observed for LBP prevalence, with the sharpest declines during 1990 to 1993, at −0.876 and −1.298 respectively; however, notably, the prevalence in the 0–19 age group increased during this period. The trend in YLDs mirrored that of prevalence.

In contrast, NP incidence rates among individuals aged ≥65 and those aged 20–65 experienced a rapid increase from 1990 to 1995, followed by a sharp decline until 2005, after which they began to rise again. For the 0–20 age group, the incidence remained generally stable, without a noticeable decrease between 1990 and 1995. Trends in NP prevalence and YLDs closely matched those of incidence. This indicates that since 2005, there has been a resurgence in NP cases, which warrants attention from health departments worldwide.

### Global heatmap of NP and LBP in working age adults

We analyzed the global distribution of low back pain (LBP) and neck pain (NP) among the working-age population in countries worldwide for the years 1990 and 2021. In 1990, North America faced more severe LBP issues, while in 2021, certain African countries saw a notable rise in LBP among the working-age population. Regarding neck pain, Africa had significant problems in 1990, whereas in 2021, high levels of NP were observed in parts of the Americas, Australia, and Asia (Supplementary Figures 1–3).

**Figure 3:**
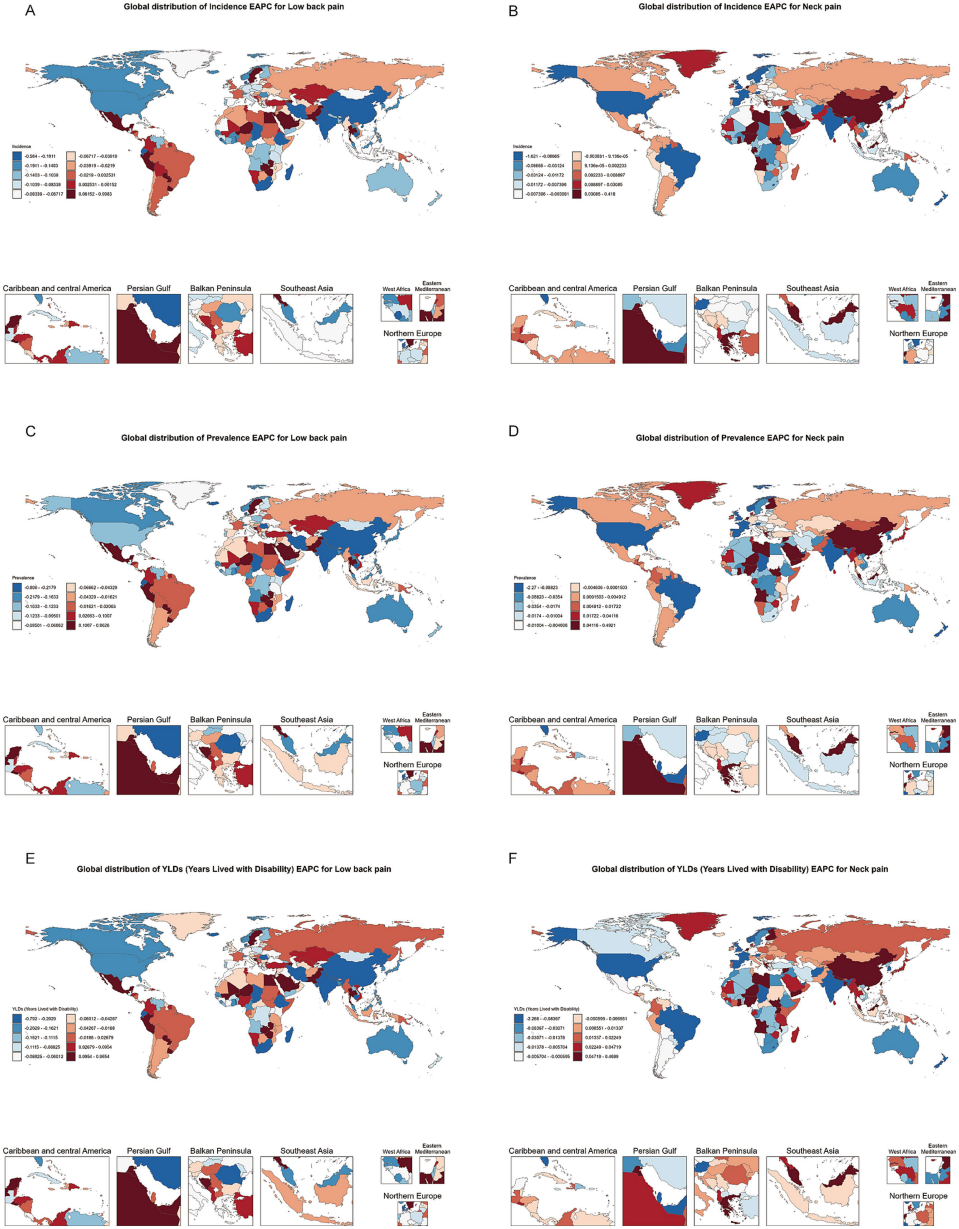
Heatmap of estimated annual percentage change (EAPC) distribution for incidence, prevalence, and YLDs of neck pain and low back pain among individuals aged 20–65 in 204 countries and regions globally from 1990 to 2021.

Between 1990 and 2021, the incidence of LBP in the working-age population increased slowly or even decreased in North America and Australia. In contrast, South America, Africa, and parts of Europe experienced a faster increase, making these regions particularly problematic. For neck pain, the global distribution was more dispersed, with some countries in Asia and Africa showing a rapid upward trend (Figure 3).

### Gender and SDI differences in work-related pain EAPC

**Figure 4:**
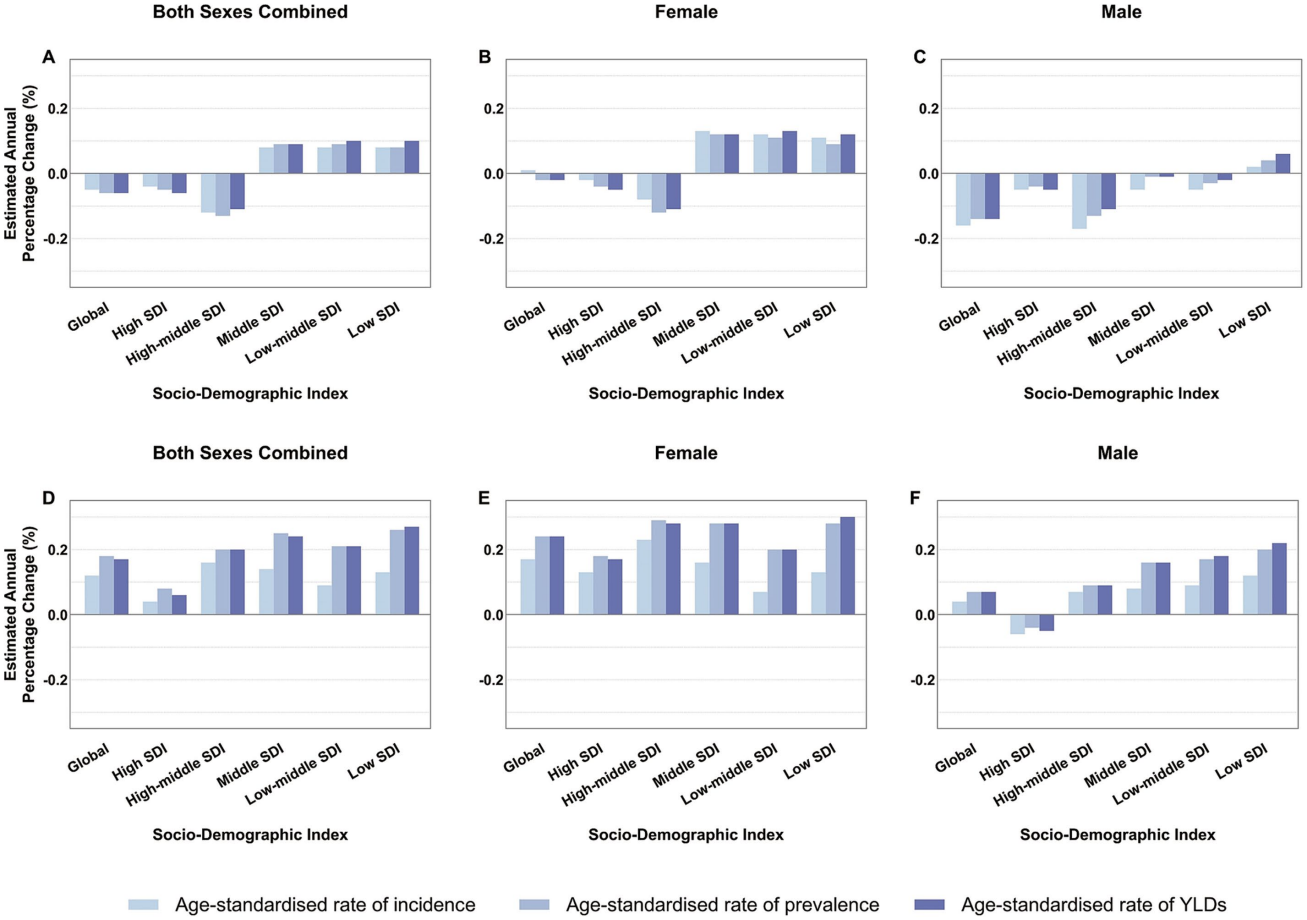
Average annual percentage change in age-standardized rates of low back pain **(A–C)** and neck pain **(D–F)** among individuals aged 20–65 globally and by SDI category from 1990 to 2021. SDI, Socio-Demographic Index; YLDs, years lived with disability.

According to the EAPC (estimated annual percentage change) bar chart analysis across different regions, we observed an increasing trend in the incidence of low back pain (LBP) in middle-income, lower-middle-income, and low-income countries, with this increase predominantly seen in the female population. In contrast, for middle-income and middle-SDI (Socio-demographic Index) countries, there was a slight decrease in LBP incidence among males (Figures 4A–C).

Regarding neck pain (NP), from a global epidemiological perspective, its incidence is on the rise. The growth rate is slowest in high-SDI countries and relatively higher in low-SDI countries. Notably, across all SDI levels, the growth rate of NP incidence in females is faster than in males. In fact, in high-SDI countries, the growth rate for males is negative (Figures 4D–F). These findings highlight the need for greater attention to be paid globally to the health issues related to LBP and NP in women.

### Population dynamics, aging effects, and epidemiological factors

**Figure 5:**
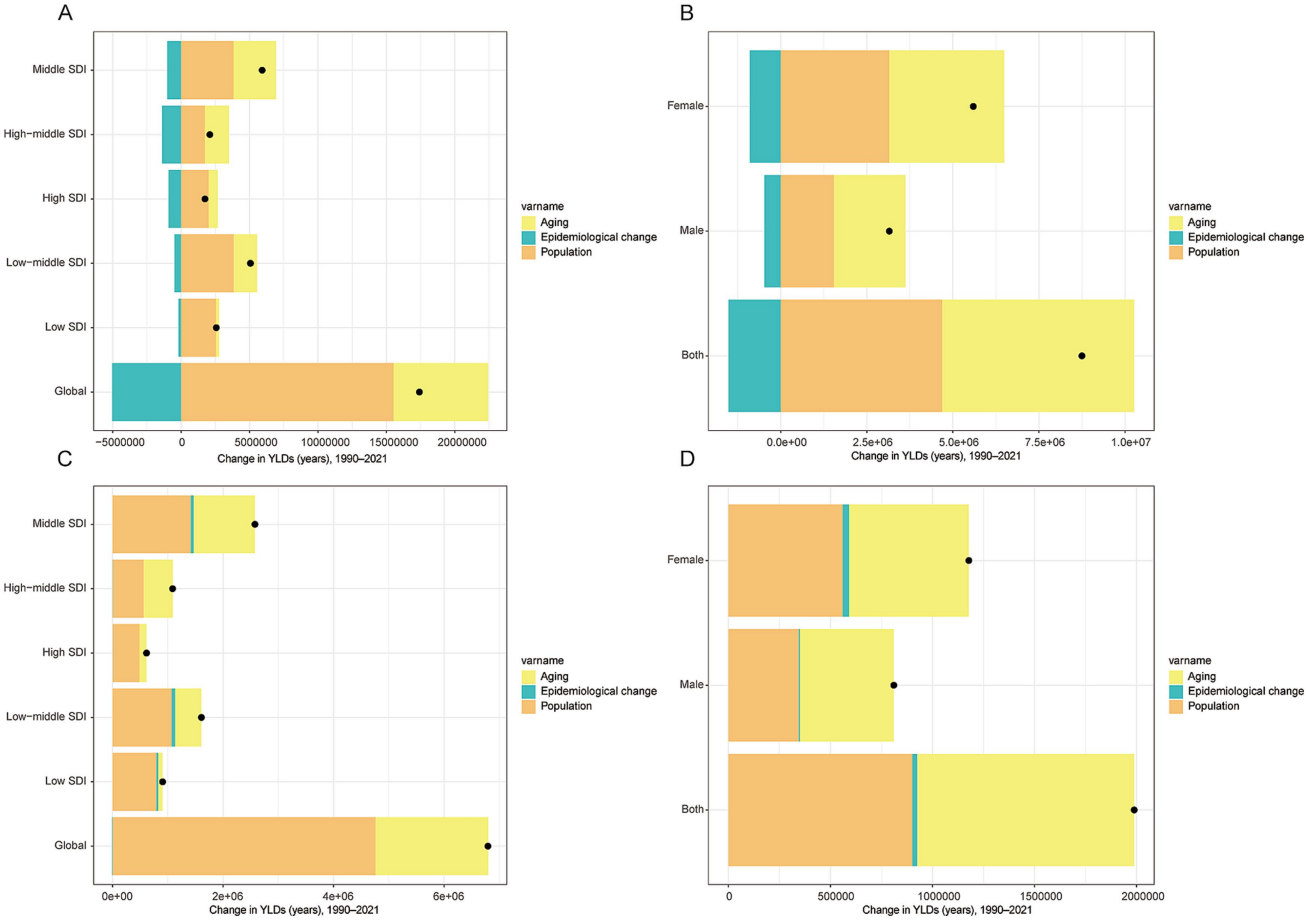
Trend decomposition analysis of Years Lived with Disability (YLDs) for neck pain and low back pain among individuals aged 20–65 from 1990 to 2021. YLDs, years lived with disability. The x-axis shows the absolute contribution to the change in total YLDs (in years) from 1990 to 2021.

Trend decomposition analysis has shed light on the multiple factors contributing to the growth of low back pain (LBP) and neck pain (NP). For LBP, population growth and aging have positively contributed to the increase in the global number of cases, with demographic factors being the primary driving force while epidemiological factors have somewhat mitigated this growth. Middle SDI countries have made the largest contribution to the global increase in LBP (Figure 5A), and the increase in female patients is greater than that in males (Figure 5B). This indicates that when considering the growth of LBP, attention must be paid not only to natural population growth but also particularly to women’s health issues.

Regarding NP, demographic factors are similarly the main driver pushing the global increase in case numbers, followed by aging factors. Similar to LBP, the impact of epidemiological factors on global changes in NP is relatively minor (Figure 5C). Notably, the growth rate of NP in females remains significantly higher than in males (Figure 5D), further emphasizing the need for greater attention to female health management in both LBP and NP.

### Health inequality and future projections

**Figure 6:**
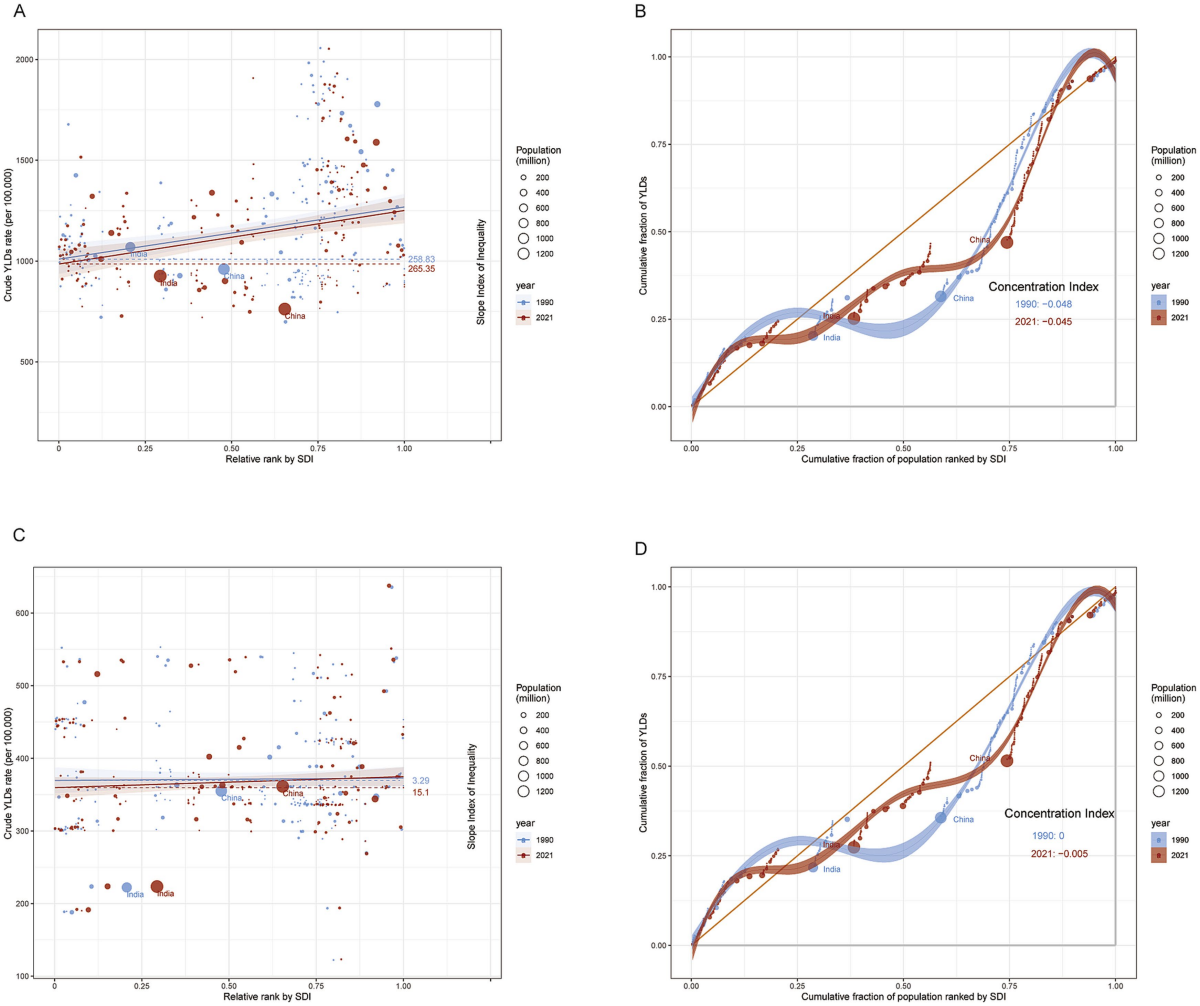
Slope Index of Inequality (SII) and Concentration Index (CI) for years lived with disability (YLDs) due to low back pain **(A,B)** and neck pain **(C,D)** among adults aged 20–65 worldwide, 1990–2021. YLDs, years lived with disability; SII, slope index of inequality; CI, concentration index. The cumulative fraction of the population ranked by SDI (x-axis) is the running share of the global population as we move up this ranking (0–1). The cumulative fraction of total YLDs (y-axis) is the running share of global YLDs contributed by those same countries (0–1). A curve near the 45° line indicates an even distribution; deviations show concentration in lower or higher SDI groups.

**Figure 7:**
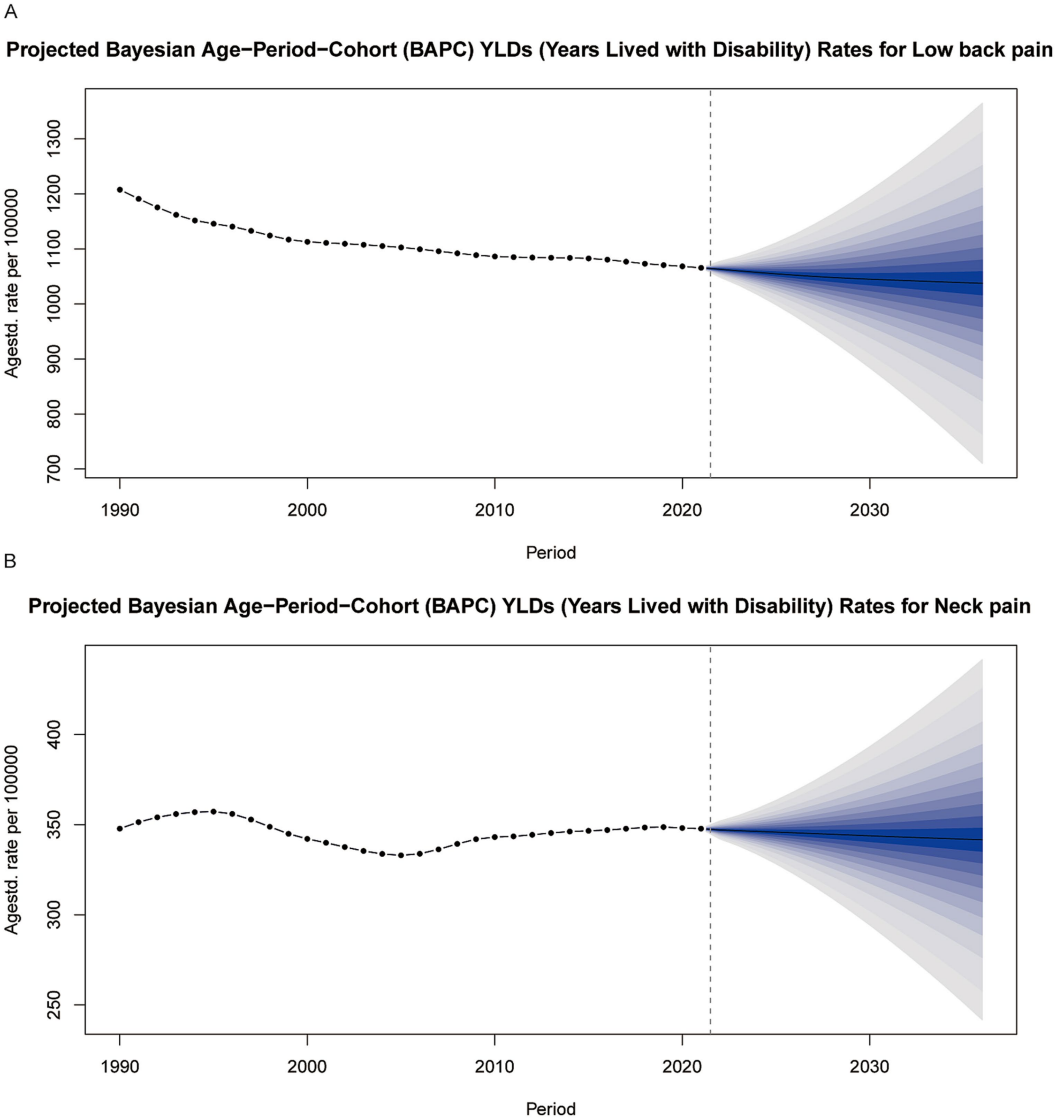
Predictions of Years Lived with Disability (YLDs) for low back pain **(A)** and neck pain **(B)** among individuals aged 20–65 from 2022 to 2036 based on the BAPC model. YLDs, years lived with disability.

Inequality index analysis reveals that as the socioeconomic level of a country improves, the incidence of low back pain (LBP) shows an increasing trend, indicating a close association between higher socioeconomic status and worse health outcomes in LBP. However, the trend for neck pain (NP) is more complex, and its relationship with the SDI is not significant (Figure 6). According to the BAPC predictive model, by 2036, the years lived with disability (YLDs) due to neck pain (NP) and low back pain (LBP) in the working-age population are expected to decrease (Figure 7).

## Discussion

Our study systematically evaluated the global burden of low back pain (LBP) and neck pain (NP) among working-age adults (aged 20 to 65) from 1990 to 2021. It revealed significant disparities in these health issues across regions, genders, and socioeconomic strata, highlighting the increasing burden and inequality driven by population aging.

Over the past three decades, while the incidence, prevalence, and years lived with disability (YLDs) for low back pain (LBP) have shown a slight decline among the global working-age population (aged 20 to 65), the corresponding metrics for neck pain (NP) have exhibited a mild upward trend (1). Specifically, the incidence, prevalence, and years lived with disability (YLDs) for low back pain (LBP) have maintained a steady decline over the past 30 years. In contrast, neck pain (NP) reached its lowest point around 2005 and has since shown a rebounding trend. These findings align with previous studies indicating an increasing burden of back and neck pain in aging societies. Additionally, this study highlights that Socioeconomic factors significantly affect the patterns of low back pain (LBP) and neck pain (NP). In developed countries, despite higher incidence and prevalence rates for LBP, the annual growth rate is slow, and YLDs are relatively low. For NP, developed countries generally have lower incidence rates, while developing countries experience higher rates. This may be due to greater risk factors in less developed regions, such as poor working conditions, lack of preventive measures, and limited healthcare access. In developing countries, socioeconomic improvements can initially increase LBP and NP incidence and prevalence before stabilizing (22). Notably, similar to previous studies, we also found that the growth rates of low back pain (LBP) and neck pain (NP) are significantly higher in females compared to males, which may be attributed to both physiological and social factors (23). Women’s higher incidence of low back and neck pain likely reflects multiple interacting mechanisms. First, endocrine differences are implicated. Evidence indicates that testosterone exerts an overall antinociceptive and protective effect: removal of endogenous testosterone prolongs pain sensitization in some models, whereas testosterone supplementation in females attenuates sensitization. By contrast, estradiol shows mixed effects on pain, with opposite actions reported across different models (24, 25). Second, anatomical and biomechanical factors may contribute: compared with men, women generally have smaller cervical vertebral dimensions, lower muscle strength, and reduced ligamentous strength, which may increase susceptibility to neck pain (26). In addition, within a biopsychosocial framework, psychological distress markedly elevates the risk of chronic conditions such as low back and neck pain. In a study by Kerstin et al., women exhibited higher levels of depressive symptoms associated with back pain, and the authors also found that psychological problems may predict the onset of depression (27). Overall, women appear more susceptible to low back and neck pain than men.

Reducing the burden of LBP and NP calls for interventions that reflect gender, regional, and socioeconomic realities. Policymakers can focus on three linked actions: (i) set low-cost ergonomic standards that workplaces can adopt even in low-SDI areas; (ii) widen subsidies for basic pain-management services so cost does not add to existing inequalities; and (iii) add gender-sensitive measures to national musculoskeletal-health programs to address the higher burden in women. At the same time, global and national health bodies should improve surveillance, paying close attention to women and to the needs of low- and middle-income countries. Tailoring efforts to local context and gender differences will make public-health strategies more effective and help ease the worldwide burden of LBP and NP.

While this study utilized the standard methods of the Global Burden of Disease (GBD), several limitations should be noted. First, in low SDI regions, particularly where diagnostic infrastructure is limited, the burden of neck pain (NP) may be underestimated (28). Second, despite standardization by GBD, regional variations in clinical definitions (such as diagnostic criteria for NP) could introduce bias (29). Third, BAPC predictions assume stable trends in risk factors, which may not account for the impacts of disruptive events like pandemics or technological revolutions (8).

Our study has several limitations. First, the observed “higher burden in high-SDI settings” may partly reflect systematic underestimation in low-SDI countries due to incomplete primary care and surveillance (limited diagnostic access, insufficient pain assessment and follow-up, underreporting), introducing measurement bias in cross-SDI comparisons. Second, we relied on country–year aggregates from the GBD and conducted analyses at the global and SDI-stratified levels rather than at individual country or subnational levels, which limits our ability to capture within-group heterogeneity related to climate zones, health-system capacity, and occupational structure. Third, BAPC projections are not well suited to detecting structural breaks caused by policy changes, shifts in diagnostic criteria, or disruptive events (e.g., pandemics, economic shocks, rapid diffusion of digital health), and any systematic error in the inputs can propagate into the forecasts.

Prospective, population-based cohort studies on LBP and NP remain scarce—most available evidence is cross-sectional or limited to single occupations (30). Rigorous implementation trials testing the real-world effectiveness and cost-efficiency of ergonomic or digital self-management interventions are likewise limited in number and scope (31). Establishing large longitudinal cohorts, conducting pragmatic trials across diverse settings, and expanding multi-omics investigations would therefore address these critical evidence gaps and guide more targeted prevention strategies.

## Conclusion

Our findings show that, although global YLDs from low-back and neck pain have declined slightly, the burden continues to rise among women and in low-income countries. This disparity highlights the pivotal role of socioeconomic and gender factors in musculoskeletal health. Policymaking should therefore priorities these high-burden groups, emphasize low-cost ergonomic solutions and protections for female workers, and embed equity-focused principles into occupational-health planning to curb further increases in LBP and NP.

## Data Availability

The original contributions presented in the study are included in the article/Supplementary material, further inquiries can be directed to the corresponding author.

## Footnotes

^1^ https://ec.europsa.eu/eurostat/

^2^ https://ghdx.healthdata.org/gbd-2021
