# Supplementary figures and images for: Global trends in low back pain and neck pain in the working population: implications for occupational health
Source: Front Public Health. 2025 Sep 15;13:1605072. doi: 10.3389/fpubh.2025.1605072 (PMC12478301; doi:10.3389/fpubh.2025.1605072)

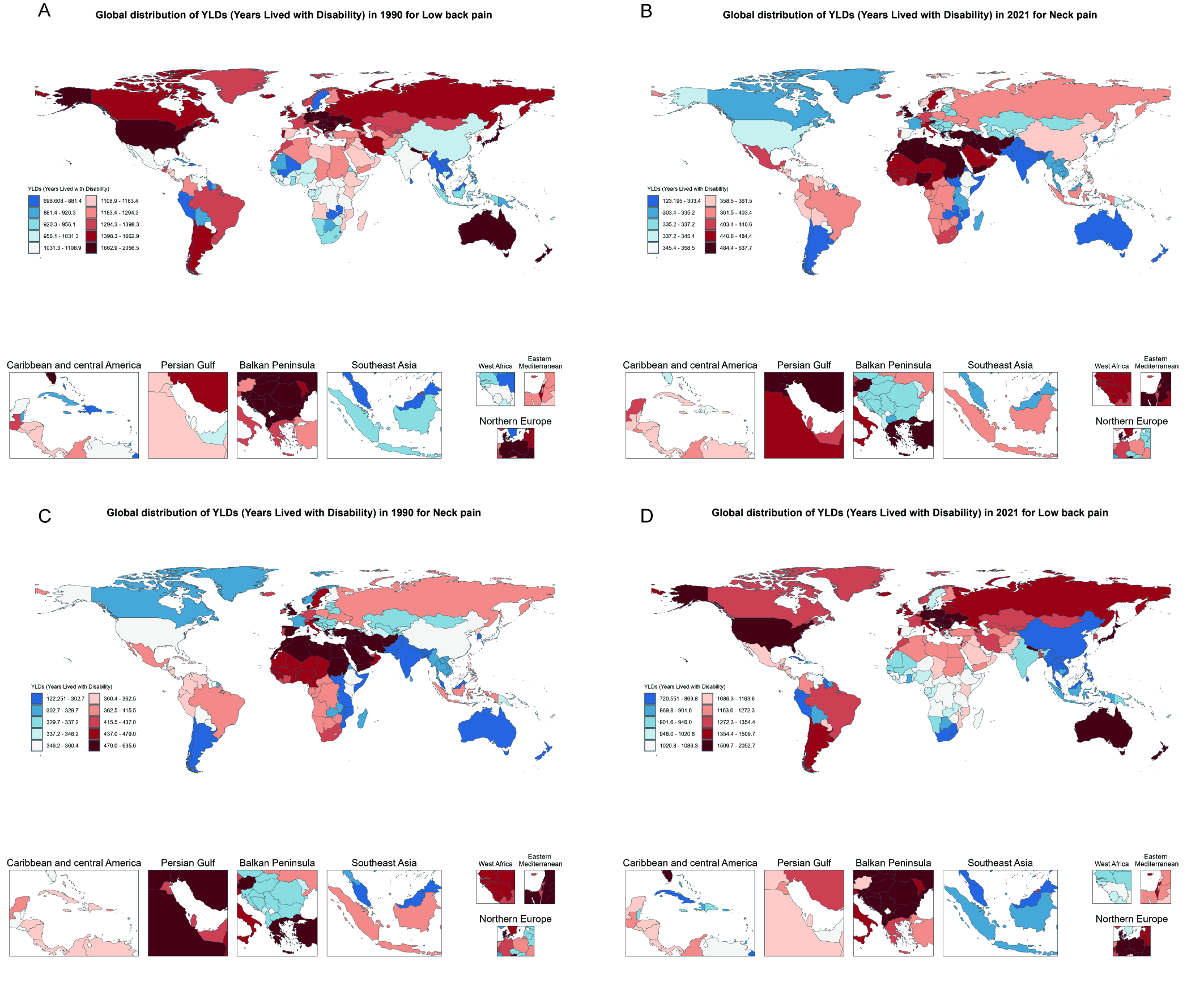

Supplement: Supplementary file 1 [file Image_1.tif]

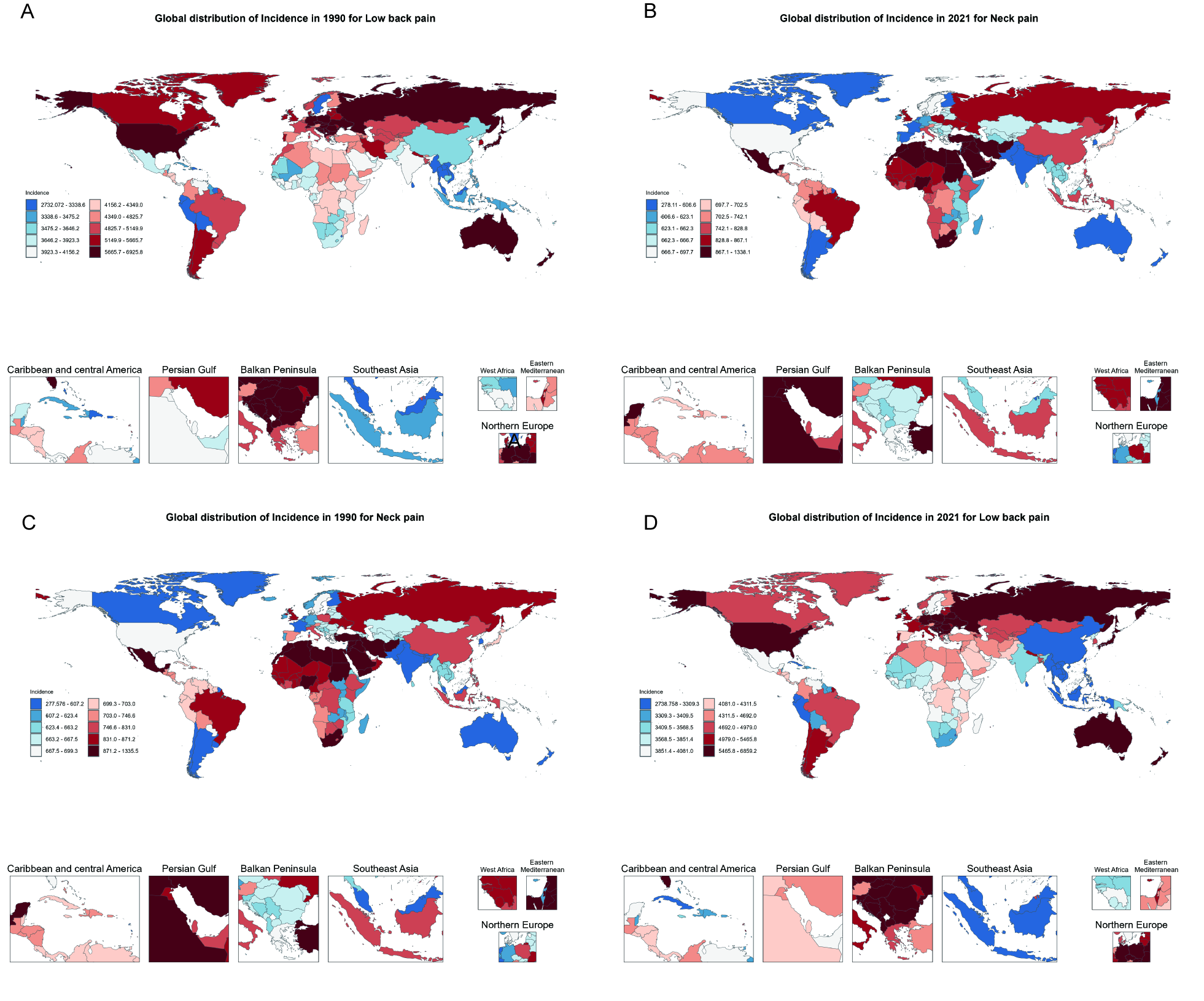

Supplement: Supplementary file 2 [file Image_2.tif]

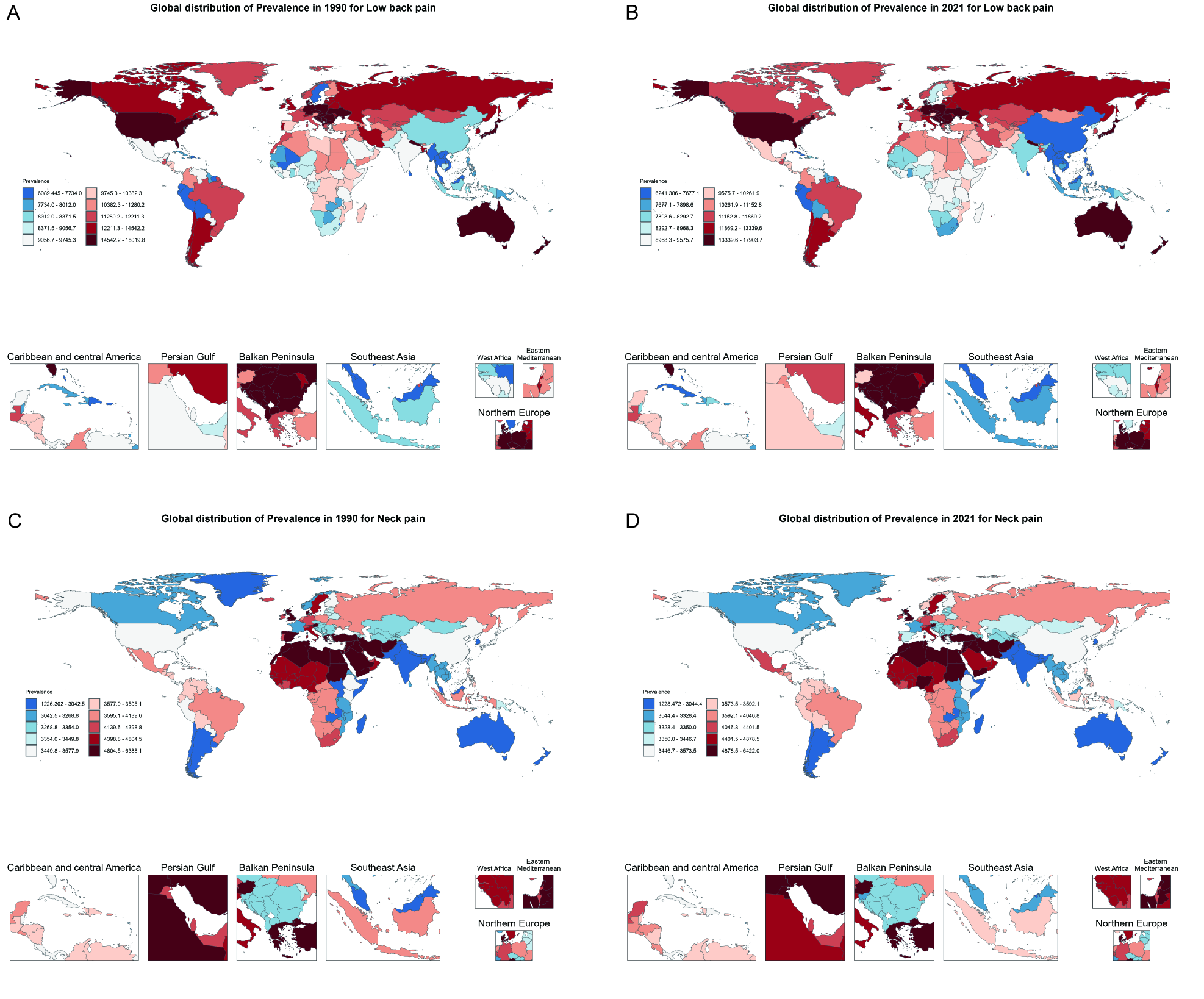

Supplement: Supplementary file 3 [file Image_3.tif]
